# Influences of different developmental periods of taurine supplements on synaptic plasticity in hippocampal CA1 area of rats following prenatal and perinatal lead exposure

**DOI:** 10.1186/1471-213X-7-51

**Published:** 2007-05-19

**Authors:** Shan-Shan Yu, Ming Wang, Xin-Mei Li, Wei-Heng Chen, Ju-Tao Chen, Hui-Li Wang, Di-Yun Ruan

**Affiliations:** 1 Department of Neurobiology and Biophysics, School of Life Sciences, University of Science and Technology of China, Hefei, Anhui 230027, PR China

## Abstract

**Background:**

Previous study has demonstrated that dietary taurine supplement protected rats from impairments of synaptic plasticity induced by postnatal lead exposure. However, little is known about the role of taurine in the presence of prenatal and perinatal lead exposure. We investigated the possible effect of taurine supplement on prenatal and perinatal lead-induced synaptic plasticity deficit and determined developmental periods critical for the effect of taurine.

**Results:**

In the present study, taurine was administrated to prenatal and perinatal lead-exposed rats in different developmental periods: from prenatal to weaning (Lead+PW-Tau), from weaning to life (Lead+WL-Tau), and from prenatal to life (Lead+PL-Tau). We examined the input-output (I/O) function, paired-pulse facilitation (PPF) and the long-term potentiation (LTP) of field excitatory postsynaptic potential (fEPSP) in the hippocampal CA1 area of rats on postnatal days 18–25 (P18–25) or days 60–75 (P60–75). We found that (1) on P18–25, taurine had no evident effect on I/O functions and PPF ratios of lead-exposed rats but caused a 12.0% increase in the LTP amplitudes of these animals; (2) on P60–75, taurine significantly elevated lead depressed I/O functions and PPF ratios in Lead+PW-Tau and Lead+PL-Tau rats, but failed in Lead+WL-Tau rats. The amplitudes of LTP of lead-exposed rats were all significantly increased by additional taurine supplement in any developmental period compared with untreated rats. Thus, taurine appeared to have the most effect during the prenatal and lactation periods and its effects on younger rats would not be manifest until the adult life; and (3) the level of lead deposition in hippocampus was evidently reduced by additional treatment of taurine in lead-exposed rats, compared with untreated rats.

**Conclusion:**

Taurine supplement can protect the adult rats from synaptic plasticity deficits following prenatal and perinatal lead exposure, and the protective effects are critical for the prenatal and lactation periods of lead-exposed rats.

## Background

Lead is a well-known environmental toxicant that induces mental impairments in children and adolescents [1-4]. Whereas the cellular mechanisms underlying the manifestation of lead-induced neurotoxicity remained elusive, alterations in the properties of glutamatergic, cholinergic, and dopaminergic neurotransmitters function and signal transduction have been reported [5,6]. Besides, it is well documented that the induction, expression, and maintenance of long-term potentiation (LTP), a principal experimental model used to study the role of synapses in learning and memory, are impaired in hippocampal dentate gyrus (DG) and CA1 regions of animals exposed to lead [7-11].

Taurine is an endogenous amino acid and is present at concentrations second only to those of glutamate in the mammalian central nervous system [12,13]. The essential roles of this amino acid in development, osmoregulation, and survival of neurons are well documented [14-16]. In addition, some studies have proposed that taurine can protect neural cells in some pathological conditions. For instance, taurine attenuates Ca^2+ ^influx and blocks the excitatory neurotoxical cascades evoked by ischemia and hypoxia [17-21]. Moreover, taurine protects against lead-induced deficits of LTP in the DG areas of rats [22].

Recently, several clinical studies investigated the effects of low-level prenatal and perinatal lead exposure on intellectual development in children and found that mater-derived lead exposure would have a more powerful and lasting impact on neurobehavioral development of offspring than postnatal exposure [23-27]. Actually, even very low level of maternal blood lead (10 μg/dL) may induce intelligence quotient changes in the child [2]. The vulnerability of the fetal brain to the lead toxicity at least partly results from the immature blood brain barrier (BBB) and the absence of tissue protein complexes that can sequester lead [28].

By using electrophysiological methods, our previous results showed that dietary taurine supplement protected rats from the impairments of synaptic plasticity induced by postnatal lead exposure [22]. However, little is known about the possible role of taurine in the presence of fetal lead exposure. In addition, no studies have evaluated the developmental periods that are critical for proper protection from synaptic plasticity deficits in the rat pups with prenatal and perinatal lead exposure. To investigate the protective role of taurine in different developmental periods and to understand how this amino acid interacts with lead, we conducted the experiments with hippocampal slices and found that taurine was an effective drug to protect rats from synaptic plasticity deficits following prenatal and perinatal lead exposure. In addition, our data indicated that taurine supplement during the prenatal and lactation periods appeared to have the most potent protection against synaptic plasticity deficits in the adult rats that had lead exposure during the fetal and neonatal age.

## Results

### Lead concentrations in hippocampus

Lead concentrations in hippocampi of the dams in different developmental periods of postnatal days 18–25 (P18–25) and days 60–75 (P60–75) are listed in Table 1 and Table 2 respectively. In both developmental periods, there were significant differences in lead concentrations between Con and Lead groups (in both periods, *p *< 0.01), but no significant differences were found between Con and Con+Tau groups (in both periods, *p *> 0.05). At the age of P18–25 days (Table 1), lead concentration in Lead+PW-Tau group was significantly decreased relative to the lead-only group, and statistically indistinguishable from that of Con group (*p *> 0.05). At the age of P60–75 (Table 2), the lead levels in hippocampi of three lead-exposed groups with additional taurine supplements were significantly lower than that of Lead group (Lead+PW-Tau: *p *< 0.01; Lead+WL-Tau: *p *< 0.05; Lead+PL-Tau: *p *< 0.01). No evident differences were found among Lead+PW-Tau, Lead+PL-Tau and Con groups (*p *> 0.05). These results demonstrate that 0.625% taurine supplement in any of developmental periods significantly reduced the concentration of lead in the brain. In Lead+WL-Tau group, however, the lead concentration was still significantly higher than that of Con group (*p *< 0.05).

**Table 1:** Lead concentration in the hippocampus of P18–25 rats

Groups	Con (n = 8)	Lead (n = 10)	Con+Tau (n = 8)	Lead+PW-Tau (n = 7)
Lead concentration in hippocampus(μg/g)	0.357 ± 0.118	1.237 ± 0.554^a^	0.480 ± 0.148	0.654 ± 0.144^b^

All the values are expressed as mean ± SEM; the number of experiments is shown in brackets (n =); ^a ^significantly different from Con group (*p *< 0.01); ^b ^significantly different from Lead group (*p *< 0.05) and statistically indistinguishable from Con group (*p *> 0.05). One-way ANOVA with the Bonferroni post hoc test were used for statistical analysis.

**Table 2:** Lead concentration in the hippocampus of P60–75 rats

Groups	Con (n = 8)	Lead (n = 8)	Con+Tau (n = 7)	Lead+ PW-Tau (n = 7)	Lead+ WL-Tau (n = 8)	Lead+ PL-Tau (n = 8)
Lead concentration in hippocampus(μg/g)	0.249 ± 0.096	1.514 ± 0.64^a^	0.249 ± 0.143	0.475 ± 0.129^b^	0.929 ± 0.094^c^	0.502 ± 0.148^b^

All the values are expressed as mean ± SEM; the number of experiments is shown in brackets (n =); ^a ^significantly different from Con group (*p *< 0.01); ^b ^significantly different from Lead group (*p *< 0.01) and statistically indistinguishable from Con group (*p *> 0.05); ^c ^significantly different from Lead (*p *< 0.01), Con (*p *< 0.01), Lead+PW-Tau (*p *< 0.05) and Lead+PL-Tau groups(*p *< 0.05). One-way ANOVA with the Bonferroni post hoc test were used for statistical analysis.

### P18–25 groups

#### Effect of taurine on I/O functions

**Figure 1:**
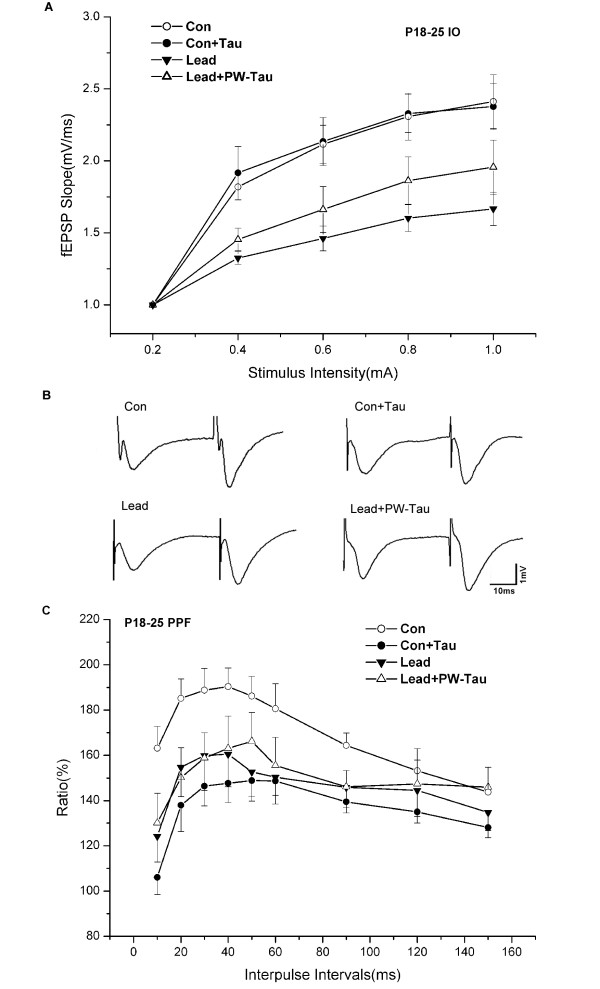
The effects of taurine on I/O functions and PPF in P18–25 groups. (A) The I/O curve was significantly depressed in Lead group compared with that of Con group (Lead: n = 9, Con: n = 11, *p *< 0.01). There was neither significant difference in I/O function between Con and Con+Tau groups (Con+Tau: n = 9, *p *> 0.05), nor a significant difference in I/O function between Lead and Lead+PW-Tau groups (n = 10, *p *> 0.05). (B) Representative traces of PPF at 40 ms ISIs in the four groups. (C) The PPF curves at varying from 10–150 ms in different groups. The average peak facilitation was significantly lower in Lead group than that in Con group (Lead: ISI = 40 ms, n = 9; Con: ISI = 40 ms, n = 11; *p *< 0.01). Moreover, the Lead+PW-Tau group showed an insignificant augment compared with the Lead group (Lead+PW-Tau: ISI = 50 ms, n = 10; *p *> 0.05), but an evident reduction versus Con group (*p *< 0.01). The peak facilitation of the Con+Tau was significantly lower than that of controls (Con+Tau: ISI = 50 ms, n = 9; *p *< 0.01). The average peak facilitation was 190.4%, 148.8%, 160.5% and 166.3% in the Con, Con+Tau, Lead and Lead+PW-Tau groups, respectively. One-way ANOVA with the Bonferroni *post hoc *test were used for statistical analysis.

To determine the effect of taurine on basal synaptic transmission in the CA1 region, we first examined the input/output (I/O) functions before the induction of LTP. The I/O curves of Con, Con+Tau, Lead and Lead +PW-Tau groups at age of P18–25 days are presented in Fig. 1A. We found that the field excitatory postsynaptic potential (fEPSP) slope was significantly depressed in Lead group compared with that of Con group (Lead: n = 9; Con: n = 11, *p *< 0.01), indicating that the mater-derived lead exposure had an effect on the baseline synaptic transmission in the CA1 area of the hippocampus. However, there was no significant difference between Con and Con+Tau groups (Con+Tau: n = 9, *p *> 0.05). We next investigated the effect of taurine on lead-exposed pups and no significant difference in I/O functions was found between Lead and Lead+PW-Tau groups (Lead+PW-Tau: n = 10, *p *> 0.05).

#### Effect of taurine on PPF

To study the action of taurine on short-term synaptic plasticity, we next examined the paired-pulse facilitation (PPF) ratio by measuring fEPSP response to two stimuli delivered at short inter-stimulus intervals (ISIs) from 10 to150 ms (Fig. 1C) and the waveform changes are depicted in Fig. 1B. As shown in Fig. 1C, the average peak facilitation was significantly lower in Lead group than that in Con group (Lead: 160.5 ± 14.4%, ISI = 40 ms, n = 9; Con: 190.4 ± 8.3%, ISI = 40 ms, n = 11; *p *< 0.01). We also found that taurine supplement with the coexistence of lead did not significantly alter the PPF ratio of Lead group at the age of P18–25 days, although it appreciably increased the peak facilitation of Lead group from 160.5 ± 14.4% to 166.3 ± 12.7% in Lead+PW-Tau group (Lead+PW-Tau group: ISI = 50 ms, n = 10; *p *> 0.05). On the other hand, the average peak facilitation of the control rats was significantly decreased from 190.4 ± 8.3% to 148.8 ± 6.8% by taurine application to the intact fetus (Con+Tau: 148.8 ± 6.8%, ISI = 50 ms, n = 9; *p *< 0.01).

#### Effect of taurine on LTP

**Figure 2:**
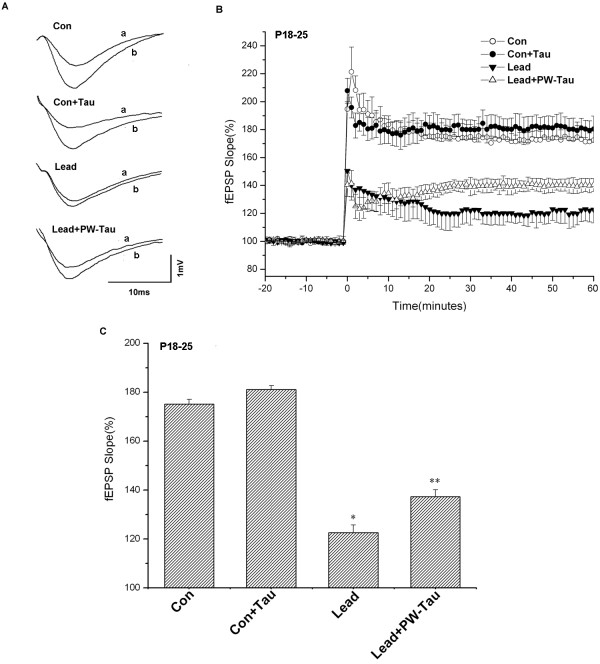
The effects of taurine on LTP induction in P18–25 groups. (A) Representative traces were recorded of field excitatory postsynaptic potentials (fEPSPs) before (a) and 60 min after (b) HFS in Con, Con+Tau, Lead and Lead+PW-Tau slices. (B) LTP was induced by stimulation at 100 Hz for 1 sec. For the sake of clarity, points are plotted every 3 min here and in subsequent LTP figures. The amplitude in the Lead group was depressed compared with that of Con group (*p *< 0.01). There was no significant difference in LTP amplitude between Con and Con+Tau groups (*p *> 0.05). The LTP amplitude in Lead+PW-Tau group was 12.0% higher than that of Lead group (*p *< 0.01) but still lower than that of controls (*p *< 0.01). (C) Histogram showing mean ± SEM percentage of fEPSP slope in 40–60 min normalized to the baseline response after HFS in Con, Con+Tau, Lead and Lead+PW-Tau subjects (Con: 175.1 ± 7.6%, n = 9; Con+Tau: 181.1 ± 8.3%, n = 8; Lead: 122.6 ± 9.6%, n = 8; Lead +PW-Tau: 137.3 ± 5.3%, n = 9). *p < 0.01, versus Con group. **p < 0.01, versus Lead group and *p*<0.01, versus Con group. One-way ANOVA with the Bonferroni *post hoc *test were used for statistical analysis.

Fig. 2 illustrates LTP in four P18–25 groups. After 20-min baseline fEPSP recording, a high frequency stimulus (HFS, 100 Hz, 1 sec) was applied to induce LTP; fEPSPs were recorded for more than 60 minutes after HFS. Fig. 2A shows the waveform alterations before and after LTP induction in P18–25 Con, Con+Tau, Lead and Lead+PW-Tau groups, respectively. Fig. 2B shows the amplitudes of LTP in these four groups. Notably, LTP was reduced to 122.6 ± 9.6% in Lead group compared with 175.1 ± 7.6% in Con group (*p *< 0.01). There was no significant change in the amplitudes of LTP between control and control with a taurine supplement groups (Con: 175.1 ± 7.6%, n = 9; Con+Tau: 181.1 ± 8.3%, n = 8; *p *> 0.05). However, significant difference was found between Lead and Lead +PW-Tau groups (Lead: 122.6 ± 9.6%, n = 8; Lead +PW-Tau: 137.3 ± 5.3%, n = 9; *p *< 0.01). In fact, with a taurine supplement throughout prenatal and lactation periods, the LTP amplitude of lead-exposed group with taurine supplement was 12.0% higher than that of the lead exposed pups without any taurine supplement (*p *< 0.01), but still lower than that of the controls (*p *< 0.01). These results suggest that, whereas taurine protected the lead-induced impairment of LTP in the CA1 area to some extent, it can not entirely reverse the lead-induced depression shortly after the coexistence with lead.

### P60–75 groups

#### Effect of taurine on I/O functions

**Figure 3:**
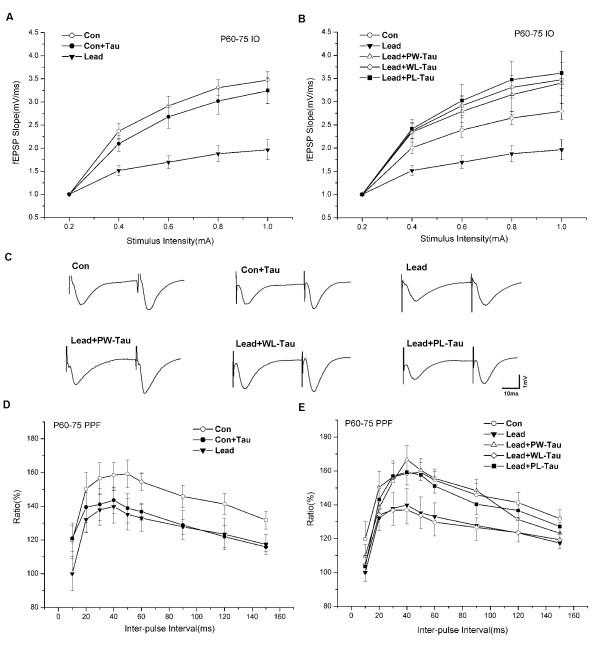
The effects of taurine on I/O functions and PPF in P 60–75 groups. (A) The fEPSP slope in Lead group was significantly decreased compared with that in Con group (Lead: n = 9; Con: n = 9; *p *< 0.01). No significant difference in I/O function was found between Con and Con+Tau groups (Con+Tau: n = 8, *p *> 0.05). (B) The I/O curves in three taurine supplemented groups were significantly increased relative to Lead group (Lead+PW-Tau: n = 9, *p *< 0.01; Lead+PL-Tau: n = 9, *p *< 0.01; Lead+WL-Tau: n = 10, *p *< 0.01). No evident differences in I/O function were found among Lead+PW-Tau, Lead+PL-Tau and Con groups (*p *> 0.05). However, the I/O curve in Lead+WL-Tau group differed significantly from that of Con group (*p *< 0.05). (C) Representative traces of PPF at 40-ms ISI in the six groups. (D) The PPF curve at varying from 10–150 ms in each group. The mean peak facilitation was 12.6% lower in Lead than that in Con group (Lead: 139.8 ± 9.7%, ISI = 40 ms, n = 8; Con: 159.2 ± 8.1%, ISI = 50 ms, n = 11; *p *< 0.01). A significant decrease of peak facilitation in Con+Tau was observed compared with Con group (Con+Tau: 143.6 ± 7.8%, ISI = 40 ms, n = 9; *p *< 0.01). (E) The peak facilitation ratios in Lead+PW-Tau and Lead+PL-Tau groups were evidently enhanced compared with the Lead (Lead+PW-Tau: 166.7 ± 8.5%, ISI = 40 ms, n = 9, *p *< 0.01; Lead+PL-Tau: 159.3 ± 7.5%, ISI = 40 ms, n = 9, *p *< 0.01), but were statistically indistinguishable from that of Con group (*p *> 0.05). In contrast, the PPF in Lead+WL-Tau group remained as low as that in Lead group (Lead+WL-Tau: 137.0 ± 8.3%, ISI = 40 ms, n = 10; Lead: 139.8 ± 9.7%, ISI = 40 ms, n = 8; *p *> 0.05) and differed significantly from that of Con group (*p *< 0.01). One-way ANOVA with the Bonferroni *post hoc *test were used for statistical analysis.

As shown in Fig. 3A, the fEPSP slope in Lead group was significantly decreased compared with that in Con group (Lead: n = 9; Con: n = 9; *p *< 0.01). This suggests that lead exposure during the prenatal and perinatal periods may induce a further depression on baseline synaptic transmission in the CA1 area. Consistent with the results obtained in younger groups, no significant difference in stimulus-response curves was found between the P60–75 Con and Con+Tau groups (Con+Tau: n = 8, *p *> 0.05), indicating that the I/O functions of grown-up control rats were not affected by taurine supplement. Fig. 3B illustrates the effects of taurine on I/O functions of three lead-exposed groups with dietary taurine supplements. Compared with that of Lead, the stimulus-response curves of fEPSP slope in these three follow-up groups were all significantly increased by taurine administrations (Lead+PW-Tau: n = 9, *p *< 0.01; Lead+PL-Tau: n = 9, *p *< 0.01; Lead+WL-Tau: n = 10, *p *< 0.01). However, no evident differences in stimulus-response curves were found among the former two groups and Con group (*p *> 0.05). As to the Lead+WL-Tau group, the stimulus-response curve was significantly lower than that of Con group (*p *< 0.05), indicating the least augment of I/O function. This result demonstrates that taurine supplement from weaning till life did not reverse the lead-induced impairment of baseline synaptic transmission.

#### Effect of taurine on PPF functions

On P60–75 days, the mean peak facilitation of lead-exposed rats was 12.6% lower than that of Con group (Lead: 139.8 ± 9.7%, ISI = 40 ms, n = 8; Con: 159.2 ± 8.1%, ISI = 50 ms, n = 11; *p *< 0.01), indicating a lasting effect of prenatal and perinatal lead exposure on short-term synaptic plasticity (Fig. 3D). We also investigated the effects of taurine on the control rats. Consistent with the results in younger groups, a significant decrease in the peak facilitation in Con+Tau was observed compared with Con group (Con+Tau: 143.6 ± 7.8%, ISI = 40 ms, n = 9; *p *< 0.01; Fig. 3D). This result suggests that, for experimental animals under normal conditions, the additional taurine intake may exert some adverse effects on synaptic facilitation. To further determine the developmental periods that are critical for protecting against lead-induced deficits in short-term synaptic plasticity, we analyzed the PPF of three follow-up groups and the PPF of fEPSP slope in Con, Lead, Lead+PW-Tau, Lead+WL-Tau and Lead+PL-Tau groups are all demonstrated (Fig. 3E). The peak facilitation ratios in Lead+PW-Tau and Lead+PL-Tau groups were evidently greater than that in Lead group (Lead+PW-Tau: 166.7 ± 8.5%, ISI = 40 ms, n = 9, *p *< 0.01; Lead+PL-Tau: 159.3 ± 7.5%, ISI = 40 ms, n = 9, *p *< 0.01), both statistically indistinguishable from that of Con group (*p *> 0.05). In contrast, the peak facilitation ratio in Lead+WL-Tau group remained as low as that in Lead group (Lead+WL-Tau: 137.0 ± 8.3%, ISI = 40 ms, n = 10; Lead: 139.8 ± 9.7%, ISI = 40 ms, n = 8; *p *> 0.05) and differed significantly from that of Con group (*p *< 0.01). Fig. 3C shows the waveform change in each group. These results demonstrate that taurine supplement from prenatal to life or from prenatal to weaning protected against the lead-induced deficits of short-term synaptic plasticity effectively. Contrarily, when it was applied to lead-exposed pups after weaning, taurine did not show such an effect.

#### Effect of taurine on LTP

**Figure 4:**
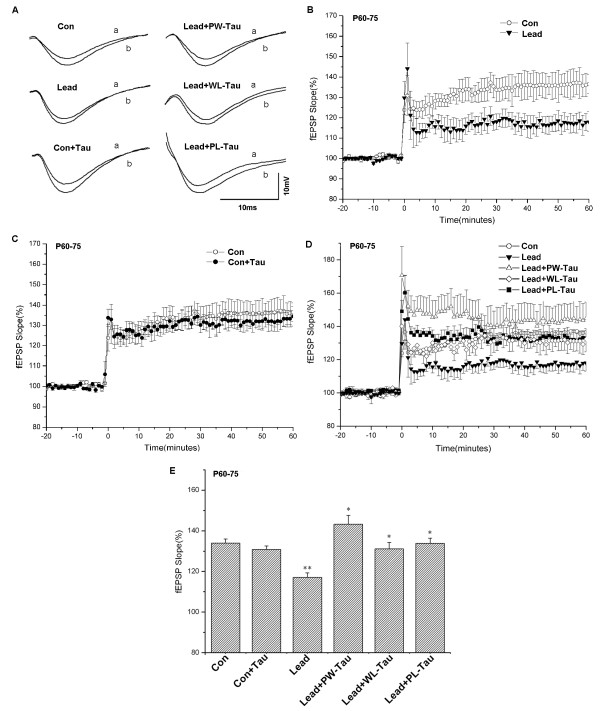
The effects of taurine on LTP induction in P60–75 groups. (A) Representative traces were recorded from P60–75 Con, Con+Tau, Lead, Lead+PW-Tau, Lead+WL-Tau and Lead+PL-Tau slices before (a) and 60 min after (b) HFS was applied. (B) The LTP amplitude in Lead group was significantly decreased relative to Con group (Lead: 117.1 ± 4.7%, n = 8; Con: 134.0 ± 5.4%, n = 9, *p *< 0.01). (C) No significant difference in LTP induction was found between Con and Con+Tau groups (Con: 134.0 ± 5.4%, n = 9; Con+Tau: 131.0 ± 4.0%, n = 8, *p *> 0.05). (D) The LTP amplitudes in the Lead+PW-Tau, Lead+WL-Tau and Lead+PL-Tau groups were all significantly increased relative to the Lead group (Lead: 117.1 ± 4.7%, n = 8; Lead+PW-Tau:143.3 ± 8.2%, n = 8; Lead+WL-Tau: 131.2 ± 5.3%, n = 10; Lead+PL-Tau: 133.9 ± 5.2%, n = 8; *p *< 0.01), but were statistically indistinguishable from that of Con group (Con: 134.0 ± 5.4%, n = 9; *p *> 0.05). Moreover, there were no significant differences in LTP induction among three lead-exposed with taurine supplement groups (*p *> 0.05). (E) Histogram showing mean ± SEM percentage of fEPSP slope in 40–60 min normalized to the baseline response after HFS in six P60–75 groups. *p < 0.01, versus Lead group, respectively. **p < 0.01, compared with Con group. One-way ANOVA with the Bonferroni *post hoc *test were used for statistical analysis.

In the experiments centered on P18–25 days, taurine failed to reverse the impaired LTP in Lead group compared with untreated group. To further determine to what extent taurine could exert its protective action and to evaluate the periods important for the protection of LTP, we followed up the lead-exposed young groups which had received taurine supplements in different developmental periods. Over the postnatal period of 60–75 days, LTP of Con, Con+Tau, Lead, Lead+PW-Tau, Lead+WL-Tau and Lead+PL-Tau groups were examined (Fig. 4B, C, D) and the waveform alterations before and after LTP induction in each group are shown in Fig. 4A.

As shown in Fig. 4B, the LTP amplitude in P60–75 Lead group was significantly decreased relative to Con group (Lead: 117.1 ± 4.7%, n = 8; Con: 134.0 ± 5.4%, n = 9, *p *< 0.01), indicating an apparent maternal lead-induced impairment of fEPSP LTP in the adult hippocampal CA1 area. Consistent with the results in P18–25 groups (Fig. 2B), there was no significant difference in LTP amplitudes between the control and control with taurine supplement group (Con: 134.0 ± 5.4%, n = 9; Con+Tau: 131.0 ± 4.0%, n = 8, *p *> 0.05; Fig. 4C).

As shown in Fig. 4D, the LTP amplitudes in the Lead+PW-Tau, Lead+WL-Tau and Lead+PL-Tau groups were all significantly increased versus Lead group (Lead: 117.1 ± 4.7%, n = 8; Lead+PW-Tau: 143.3 ± 8.2%, n = 8; Lead+WL-Tau: 131.2 ± 5.3%, n = 10; Lead+PL-Tau: 133.9 ± 5.2%, n = 8; *p *< 0.01), statistically indistinguishable from that of Con group (n = 9; *p *> 0.05). Additionally, we found that there were no significant differences in LTP amplitudes among these lead-exposed groups with varied treatments of taurine. These results indicate that taurine protected the lead-exposed rats from LTP deficits independent of administration time.

## Discussion

According to previous animal studies, even very low level of lead exposure through the mothers can be deleterious to rat pups [2]. Several clinical studies also have demonstrated that increased maternal blood lead level is significantly associated with a decrease in subsequent intelligence, attention and other brain functions in infants and adolescents [25,26,29]. Therefore, it is proposed that the fetal brain is extremely susceptible to lead toxicity [28], suggesting the most need for protection during the prenatal and lactation periods.

Recently, accumulated evidence suggests that the primary effects of lead are mediated, in part, by its oxidative damage to cell membranes [30]. Lead has been proved to induce lipid peroxidation and degradation of phospholipids with the loss of membrane integrity in lead-burdened tissues [31,32]. In addition, lead causes damage to the placenta and disrupts the structure of blood-brain barrier (BBB) in young animals [33-35]. Therefore, it may be supposed that lead-induced oxidative damage also contributes to tissue injury in the blood-placenta barrier (BPB), which restricts drug and toxic import through the placenta to the fetus [36]. The increased permeability will then consequently facilitate the accumulation of lead to the brain [35].

Our results reveal that taurine cumbers the accumulation of lead in the brain and acts most effectively during the prenatal and lactation periods. These suggest a possible membrane-protective action of taurine, on either BBB or BPB, counteracting entry of lead into the fetal brain. This idea has been supported by the findings that taurine reinforces BBB through intensifying the integration of membrane [37-39] and that taurine intercalates into the cell membrane, thereby stabilizing it [40]. Taurine also acts on the permeability of cell membrane to the ions [40,41]. Furthermore, the antioxidant effect of taurine has been involved in the protection against lead-induced oxidative damage to the cell membrane. In both culture cells and rats, lead-induced abnormal augment of lipid peroxidation is found to be decreased following taurine supplementation. The membrane susceptibility to lipid peroxide formation therefore seems to decline [42]. Unfortunately, the direct contribution of taurine in BPB protection lacks solid evidence, whereas taurine uptake activity in syncytiotrophoblast cells might indicate some related functions [36]. An alternative explanation for the membrane-protective effects of taurine has been proposed based on taurine-zinc interactions which are closely related to development of structures such as the hippocampal formation [43]. Taurine and zinc have been found to stabilize the membrane and protect against lipid peroxidation in the retina [44,45]. Moreover, a positive correlation of zinc and taurine has already been observed in the hippocampus [43].

PPF, a short-term synaptic plasticity [46,47], has been shown to be associated with increases in presynaptic Ca^2+ ^levels and transmitter release [48-50]. In the past two decades, the residual calcium hypothesis [47] was used traditionally to explain PPF of central synaptic transmission [51-54]. Ever since, the presynaptic residual Ca^2+ ^as the sole mechanism for PPF has been questioned [55,56]. Recently, many studies have agreed that a complete understanding of the mechanisms of PPF should include that the increases of transmitter release (induced by residual Ca^2+^) generate PPF and the responsivenesses of α-amino-3-hydroxy-5-methyl-4-isoxazolepropionic acid (AMPA) receptors (modified by postsynaptic Ca^2+ ^and Ca^2+^/calmodulin (CaM) signaling pathways) regulate PPF magnitude [57-59]. Moreover, in CA1 neurons, the activities of CaM-KII and protein kinase C (PKC) are also required in the PPF resulting from postsynaptic changes [60,61]. In the present study, lead exposure significantly decreased the PPF in CA1 area. This impairment may attribute to several toxic effects of lead. First, lead blocks voltage-dependent calcium channels and then reduces the calcium influx [62]; second, lead changes Ca^2+^/CaM-mediated neurotransmitter release [63]; and third, lead can antagonize the activity of PKC at nanomolar concentrations [64].

In line with the experiments on postnatal lead-exposed animals [22,65], our results suggest that prenatal and perinatal lead exposure impairs LTP induction. LTP, as a measure of synaptic plasticity, is regarded as the neuronal basis of learning and memory. In hippocampal CA1 area, the induction of LTP could involve Ca^2+ ^influx through activated voltage-dependent Ca^2+ ^channels that follows N-methyl-D-aspartate (NMDA) receptor activation [66-69]. Furthermore, Ca^2+ ^release from intracellular stores also appears to be required to generate LTP [70,71]. Lead, the recognized nerve poison, is known to disturb calcium homeostasis by interfering with not only the extracellular Ca^2+ ^influx but also the Ca^2+ ^release and uptake of intracellular calcium stores [72]. Lead-induced oxidative damages to critical biomolecules like lipids, proteins and DNA have also been observed [73]. In addition, the lead-glutamatergic interactions have been proved by numerous electrophysiological, biochemical and behavioral studies [74-76].

When taurine was administrated to the lead-exposed animals in the present study, several mechanisms may be involved in the protection against lead-induced deficits in synaptic plasticity. First, as stated above, taurine seems to have a promising antioxidant effect against oxidative damages induced by lead [42]. In lead-poisoned rats, taurine is able to increase glutathione level, reduce oxidative stress and elevate the activity of superoxidate dismutase, contributing to the protection of brain [30]; second, taurine is a known regulator of calcium homeostasis. Under pathologic conditions, such as ischemia and seizure, taurine attenuates Ca^2+ ^influx, and therefore counteracts the elevation of intracellular Ca^2+ ^concentration [77,78]. Additionally, taurine may directly act on the sarcoplasmic reticulum and regulate Ca^2+ ^release and uptake of intracellular stores [79]; third, taurine potentiates presynaptic NMDA receptors, consequently facilitating the excitability of Schaffer-collateral fibres in rat hippocampal slices [80]; and finally, taurine is proposed to regulate the activity of NMDA receptors by stimulating N-[1-(2-thienyl)cyclohexyl]-piperidine (TCP) binding with phencyclidine-binding (PCB) sites on the receptors [81].

Taurine, as we described earlier, has multiple functions in the mammalian brain participating in volume regulation [82], neuromodulation [83] and neural cell protection [21], and it is also essential for neurodevelopment and survival for neural cells [14,16]. Considering the complex effects of this amino acid, it is hard for us to predict its behaviors when taurine coexists with lead in immature brains. In the present study, confusingly, we found the behaviors of taurine were diverse between P18–25 and P60–75 Lead+PW-Tau rats, which had received taurine supplementations identically. Indeed, the protective role of taurine manifested itself only in the older group. To explain this discrepancy, one scenario we would like to entertain is that when taurine and lead simultaneously exist in the immature brain, its manipulations are complex. Besides hampering the accumulation of lead in the brain, taurine can exert various neuroprotective actions simultaneously. On the other hand, taurine affects the fetal brain from multiple aspects, including the interactions with inhibitory amino acid neurotransmitter receptors. It has been reported that taurine may act as endogenous agonist at glycine receptors [84] and activates γ-aminobutyric acid type A (GABA_A_) receptors in immature hippocampus [85]. Having been withdrawn from the simultaneous exposure of taurine and lead, the grow-up rats in the older group thus could recover their losses in synaptic plasticity gradually. However, given that the receptors changed dramatically during the development in the rat hippocampus [86], the exact mechanism of taurine in fetal brain is beyond certainty.

Definitely, taurine exposure from prenatal to life depressed the PPF in the intact rats. However, cautions needs to be exercised when interpreting this attenuation as a decrease in presynaptic release probability, because postsynaptic changes may also contribute to the alteration in PPF [59]. Despite this problem, we speculate that an indirect inhibitory effect of taurine in immature brains, via its adverse interactions with GABAergic neurotransmission at high concentrations [87], might partially account for this change under basal conditions. This possibility, however, needs to be further assessed with more specific procedures. In addition to this effect on PPF, other negative effects at high taurine concentrations have also been identified [88,89]. Therefore, even though taurine acts protectively under various pathological conditions, taken together, its negative influences may lead to an assumption that application of excessive taurine to the intact rats might be toxic.

On the other hand, it is noteworthy that the depression happened to PPF occurred to neither LTP induction nor I/O function under basal condition. This discrepancy might be caused by a homeostasis mechanism regulated by taurine. Taurine is a powerful regulator of calcium homeostasis under conditions of low level of [Ca^2+^]_i_, as well as Ca^2+ ^overload [90] and it may up-regulate the activity of NMDA receptors [81]. It is now well established that, in hippocampal CA1 area, the LTP induction at Schaffer-collateral pathway is mainly due to the postsynaptic mechanism depending on the activation of NMDA receptors and an increase in postsynaptic Ca^2+ ^concentration [91]. I/O function, however, reflects not only the level of presynaptic neurotransmitter release but also the postsynaptic receptor response. Hence, we hypothesize that taurine may act positively at postsynaptic sites during synaptic plasticity via enhancing Ca^2+ ^concentration or receptor response, thereby counteracting the inhibition of presynaptic (or postsynaptic) neurotransmission. However, considering the limited understanding about the impact of prolonged enhanced taurine levels on synaptic functions, especially in immature brain, the mechanism of this discrepancy remains to be elucidated.

## Conclusion

In conclusion, taurine supplemented diet protects rats against synaptic plasticity deficits following prenatal and perinatal lead exposure. Most importantly, in this study, taurine appears to have the most potent effect when it is supplemented during the prenatal and lactation periods. This suggests that prenatal and lactation periods are the critical developmental periods for the protective action of taurine. In developing countries, the pollution of lead turns into a severe problem which emphasizes the proper protection of taurine in the pregnant woman. Our data also suggest the time window and dosage of taurine supplement should be considered in the future community intervention.

## Methods

### Experimental animals

The experiments were conducted on the offspring of six groups of pregnant Wistar rats (70–80 days of age, 120–200 g): control (Con), control with taurine diet (Con+Tau), lead exposure (Lead), and three groups of lead exposure dams with taurine diet during different developmental periods (Lead+PW-Tau, Lead+WL-Tau and Lead+PL-Tau). In the former two groups, the pregnant dams received either distilled water or water with 0.625% taurine from gestational day 1, with their offspring switched to the same tap water (Con) or solution (Con+Tau) at weaning on postnatal day 21. The latter four groups were lead exposed via administration of 0.2% (1090 ppm) solution of lead acetate in distilled drinking water to pregnant dams from gestational day 1 through weaning on postnatal day 21. Thus, the offspring acquired lead only through their mothers. Concomitantly, 0.625% taurine [92] were applied to Lead+PW-Tau, Lead+WL-Tau and Lead+PL-Tau groups through drinking water (20 ml per day) from prenatal to weaning, from weaning to life, and from prenatal to life respectively.

Depending on developmental stage, the dams in Lead+PW-Tau and Lead+PL-Tau groups were divided into two subgroups for extracellular recording in CA1 area of hippocampus. One subgroup was tested at the age of P18–25 days and the other at P60–75 days. The pups at P18–25 days in Lead+PW-Tau and Lead+PL-Tau groups were administrated with lead and taurine almost in the same manner (dosage and exposition phase). Hence, the data in P18–25 Lead+PL-Tau group are not presented. For any given experimental measure, equal numbers of females and males were recruited and no more than two pups were sampled from the same litter. All the animals were maintained on NIH-07 chow (Ziegler Bros., Gardners, PA) to ensure consistent levels of mineral intake (see Table 3 for the treatment of each group).

**Table 3:** Treatment of each group

	P18–25 Groups (Postnatal 18–25 days)		P60–75 Groups (Postnatal 60–75 days)	
	
	Drug(s)	Exposition phase	Drug(s)	Exposition phase
Con	-^a^		-^a^	
Con+Tau	Taurine	P-W	Taurine	P-L
Lead	Lead acetate	P-W	Lead acetate	P-W
Lead+PW-Tau	Lead acetate	P-W	Lead acetate	P-W
	Taurine	P-W	Taurine	P-W
Lead+WL-Tau	-^b^		Lead acetate	P-W
			Taurine	W-L
Lead+PL-Tau	Lead acetate	P-W	Lead acetate	P-W
	Taurine	P-W	Taurine	P-L

"P-W" represents "from prenatal to weaning"; "W-L" represents "from weaning to life"; "P-L" represents "from prenatal to life"; – ^a ^untreated with any drugs; – ^b ^will not be examined until postnatal day 60–75.

### Hippocampus lead determination

Hippocampal lead determinations were made in littermates of the animals utilized for electrophysiology on the day that recordings were made. After decapitation, two hippocampi were collected. Lead concentrations were measured by a plasmaQuad3 plasma mass spectrograph (VG Elemental, UK) after the tissues were digested with an organic tissue solubilizer.

### Slice preparations and drugs

Care of animals and experiments were conducted in accordance with the National Institutes of Health Guideline for the Care and Use of Laboratory Animals. Efforts were made to minimize the number of animals used. P18–25 (40–50 g) and P60–75 (100–180 g) rats were anaesthetized with anhydrous diethyl ether and killed by decapitation. Then the brain was quickly removed and immersed in ice-cold artificial cerebrospinal fluid (ACSF) saturated with 95% O_2_/5% CO_2 _and containing 124.0 mM NaCl, 3.0 mM KCl, 1.25 mM KH_2_PO_4_, 26.0 mM NaHCO_3_, 2.0 mM MgSO_4_, 2.5 mM CaCl_2_, and 10.0 mM glucose. Hippocampus was isolated and cut into 400 μM thick transversal slices by a manual chopper. Before recording, the slices were placed in an interface holding chamber where they were maintained in oxygenated ACSF at room temperature (21–25°C). After at least 1 hour, a slice was transferred to a submersion-type recording chamber (BSCHT Medical Systems, USA), in which it was superfused continuously with 30–32°C oxygenated ACSF at the rate of 1 ml/min. Drugs used in the present experiments were all purchased from Sigma-Aldrich, USA.

### Recording

To obtain evoked synaptic responses in CA1 area, Schaffer collateral-commissural fibers were stimulated with electrical pulses (0.1–0.6 mA, 0.2 ms, 0.05 Hz), applied through bipolar microelectrodes located in stratum radiatum. Evoked extracellular field potentials (fEPSPs) were recorded with low resistance glass micropipettes filled with 2 M NaCl (resistance 1–2 MΩ). At the start of each experiment, a full input-output (I/O) curve was constructed. The stimulus intensities that yielded 1/2 and 2/3 of the maximal response were selected for baseline measurements. PPF was tested by applying two pulses at different intervals of 10–150 ms. After recording stable baseline fEPSP responses for 20 minutes, LTP was induced by a high-frequency stimulation (HFS; 100 Hz for 1 sec). After that, testing with single shocks was continued for at least 60 minutes. The amplitudes of LTP were calculated by averaging the percentage of post-tetanus data in 40–60 minutes compared with pre-tetanus baseline data. The PPF values were calculated as EPSP2/EPSP1.

### Data analysis

Data were recorded using Igor Pro 4.05 software (Wave Metrics, OR, USA) and analyzed with Origin 7.5 (OriginLab, MA, USA). The time scale in each experiment was converted to time from the onset of the HFS. Results were expressed as means ± SEM, n represents the number of animals that were sampled. One-way ANOVA was performed to determine whether there were significant differences followed by Bonferroni test as post hoc analysis; *p *< 0.01 indicated a significant difference.

## Abbreviations

ACSF, artificial cerebrospinal fluid; AMPA, α-amino-3-hydroxy-5-methyl-4-isoxazolepropionic acid; BBB, blood-brain barrier; BPB, blood-placenta barrier; CaM, Ca2+/calmodulin; DG, dentate gyrus; fEPSP, field excitatory postsynaptic potential; GABA_A_, γ-aminobutyric acid type A; HFS, high frequency stimulus; I.C.V, intracerebroventricular; I/O, input-output; ISIs, inter-stimulus intervals; LTP, Long-term potentiation; NMDA, N-methyl-D-aspartate; PKC, protein kinase C; PPF, paired-pulse facilitation.

## Authors' contributions

SSY participated in the design of the study, carried out the electrophysiological studies, the statistical analyses and the redaction of the manuscript. MW and JTC participated in the design of the study and the final version of the manuscript. XML and WHC participated in the hippocampus lead measurements. HLW helped to prepare the experimental animals. DYR coordinated the study, provided critical review of the results and manuscript contents, and acquired the funding for the research program. All authors read and approved the final manuscript.
